# Development of a context model to prioritize drug safety alerts in CPOE systems

**DOI:** 10.1186/1472-6947-11-35

**Published:** 2011-05-25

**Authors:** Daniel Riedmann, Martin Jung, Werner O Hackl, Wolf Stühlinger, Heleen van der Sijs, Elske Ammenwerth

**Affiliations:** 1 Institute of Health Informatics, UMIT - University for Health Sciences, Medical Informatics and Technology, Eduard Wallnöfer-Zentrum I, Hall in Tirol, Austria; 2 Department for Public Health and HTA, UMIT - University for Health Sciences, Medical Informatics and Technology, Eduard Wallnöfer-Zentrum I, Hall in Tirol, Austria; 3 Department of Hospital Pharmacy, Erasmus University Medical Centre, P.O. Box 2040, 3000 CA Rotterdam, Netherlands

## Abstract

**Background:**

Computerized physician order entry systems (CPOE) can reduce the number of medication errors and adverse drug events (ADEs) in healthcare institutions. Unfortunately, they tend to produce a large number of partly irrelevant alerts, in turn leading to alert overload and causing alert fatigue. The objective of this work is to identify factors that can be used to prioritize and present alerts depending on the 'context' of a clinical situation.

**Methods:**

We used a combination of literature searches and expert interviews to identify and validate the possible context factors. The internal validation of the context factors was performed by calculating the inter-rater agreement of two researcher's classification of 33 relevant articles.

**Results:**

We developed a context model containing 20 factors. We grouped these context factors into three categories: characteristics of the patient or case (e.g. clinical status of the patient); characteristics of the organizational unit or user (e.g. professional experience of the user); and alert characteristics (e.g. severity of the effect). The internal validation resulted in nearly perfect agreement (Cohen's Kappa value of 0.97).

**Conclusion:**

To our knowledge, this is the first structured attempt to develop a comprehensive context model for prioritizing drug safety alerts in CPOE systems. The outcome of this work can be used to develop future tailored drug safety alerting in CPOE systems.

## Background

### Medication errors and adverse drug events (ADEs)

"*Errare humanum est - To err is human*". This famous quotation, derived from Cicero and Seneca the Elder, is universally applicable, including medicine. Medication errors have been identified to be a common type of medical errors [1]. The Institute of Medicine reports that a hospital patient can expect to be subject to at least one medication error per day [2].

Medication errors and adverse drug events (ADEs) can occur during every step of the medication use process [2-4]. The Council of Europe defines a medication error as *"any preventable event that may cause or lead to inappropriate medication use or patient harm while the medication is in the control of the health care professional, patient or consumer" *[3] (p.7) and an ADE as "*any injury occurring during the patient's drug therapy and resulting either from appropriate care or from unsuitable or suboptimal care" *[3] (p.1). ADEs associated with a medication error are considered to be preventable ADEs [2,3].

A further breakdown of medication errors into the five stages of the medication use process, which are prescribing, transcribing/documenting, dispensing, administering, and monitoring, allows the identification of error-prone steps. 39% [1] of all medication errors and 56% [5] - 71% [6] of preventable ADEs occur in the prescription phase.

### CPOE to prevent medication errors and ADEs

There is evidence for the effectiveness of computerized physician order entry (CPOE) in hospital settings in reducing medication errors as well as ADEs [2,7-10]. CPOE systems can be equipped with further clinical decision support (CDS). Kuperman et al. distinguishes between basic (e.g. offers drug-drug interaction checking) and advanced (e.g. includes advanced guidance for laboratory testing) medication-related decision support [11]. A systematic review performed in 2008 reported a higher relative risk reduction of medication errors of systems with advanced CDS compared to CPOE systems with limited or no CDS [8].

### CPOE and the challenge of alert fatigue

When a CPOE system is equipped with CDS, the burden of alert-handling has to be considered. As research shows, drug safety alerts as well as reminders from CDS systems are often disregarded by the prescribers. A review paper in 2006 reported alert override-rates of 49% to 96% [12]. Especially drug-drug interaction (DDI) and drug-allergy checking suffers from low specificity due to too many false positive warnings which results in high override-rates [11,12]. However, in general, all types of CDS-triggered warnings are frequently overridden [12].

Alert fatigue is a frequent complaint about CPOE systems with CDS [12-14]. An increasing number of drug safety alerts has the potential for user desensitization [15]. This desensitization leads to an override of both important (even highly important) and unimportant warnings [12]. In this work we focus on the definition of van der Sijs [16] who describes alert fatigue as the mental state that is the result of alerts consuming too much time and mental energy, which can cause relevant alerts to be unjustifiably overridden along with clinically unimportant ones. Synonyms used for the term alert fatigue are 'cry wolf syndrome' and 'pop-up fatigue' [15].

A systematic review by Khajouei et al. of the design aspects of CPOE systems reported eight studies investigating the impact of specificity, sensitivity, unclear information content, and timing of warnings on creating conditions for medication errors [14]. This review showed that low alert specificity/sensitivity and unclear information content can induce alert fatigue.

### A context-aware CPOE system

Suggestions to improve the specificity of drug safety alerts such as patient-tailored drug safety alerts can be embraced by the term 'context'.

In computer science, 'context' refers to the idea of systems sensing and reacting based on their environment. Within this work, the following definition from the area of ubiquitous computing is used: *"Context is any information that can be used to characterize the situation of an entity. An entity is a person, place, or object that is considered relevant to the interaction between a user and an application, including the user and applications themselves" *[17] (p.3). A system using 'context' tries to make assumptions about the current situation and circumstances. Dey further designates a system as a context-aware system *"if it uses context to provide relevant information and/or services to the user, where relevancy depends on the user's task" *[17] (p.4). According to this declaration, context-aware CPOE systems provide:

• **Relevant information**: for example, context-aware CPOE may prioritize drug safety alerts in order to present them in an adequate manner (life-threatening alerts interrupt the prescribing process of the user and cannot be ignored; in contrary, less relevant alerts are not interrupting the user)

• **Relevant services**: for example, context-aware CPOE may give reminders for regular laboratory monitoring or may offer drug dosing support.

**Figure 1:**
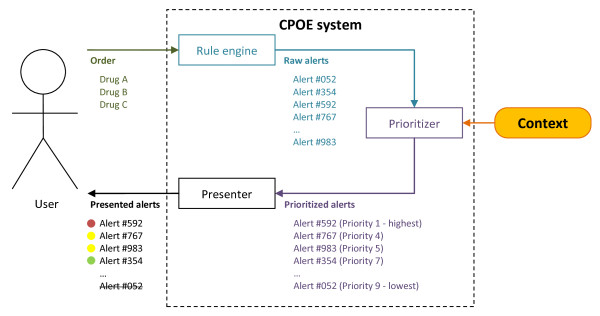
**A context-aware CPOE system**. Depending on the prescription, the rule engine of the CDS system generates raw alerts (e.g. drug-drug interaction between acetylsalicylic acid and an anticoagulant). These raw alerts are then prioritized based on context information (e.g. the dose, the age of the patient, any co-medication, or information on user or clinical department). Afterwards, the alerts are presented differently to the user according to their priority (e.g. life-threatening alerts interrupt the prescribing process and cannot be overridden).

Figure 1 schematically shows the concept of drug prescription within a CPOE system that prioritizes and presents drug safety alerts depending on the clinical context.

The European PSIP project (Patient Safety through Intelligent Procedures in medication, http://www.psip-project.eu) develops, among others, innovative and CDS systems to increase medication safety during prescription. In the PSIP project, the frequency of ADEs has been analysed for individual medical departments or hospitals. Based upon this knowledge, a rule-based CDS system has been developed [18]. However, so far it has not been exhaustively studied which possible context factors could be used to improve the specificity and sensitivity of drug safety alerts.

### Study question

• What are possible factors that can help to prioritize and present drug safety alerts in CPOE systems according to the given context?

## Methods

**Figure 2:**
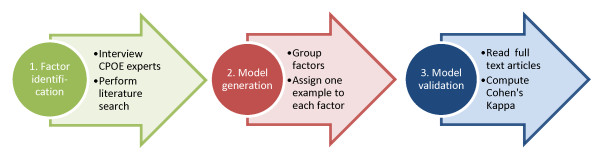
**Major phases in the development and validation of the context model**.

The methods used during the creation of the context model can be grouped into three major parts: a factor identification phase, a model generation phase and a model validation phase (see Figure 2).

### Phase 1: Identification of context factors

We combined a literature search with expert interviews to identify the possible context factors that might be used to prioritize and present drug safety alerts.

### Literature search

The literature search aimed at identifying articles dealing with contextualization or the improvement of drug safety alerting in CPOE systems. Two different search strategies were applied:

First, as a starting point, a **hand search **was performed to extract ideas for contextualized drug safety alerts in CPOE systems which focused on ten journals in the field of health informatics (for a list of all hand-searched journals see additional files 1). Two researchers (MJ and DR) carried out this search at the end of February 2010. References of the found papers were followed in order to identify further papers. The researchers also retrieved related articles of found papers, as identified by this function in PubMed.

Second, a **PubMed search **was conducted comprising two search queries (see Table 1):

**Table 1:** Search terms for the factor extraction phase

Specific PubMed search (A)		Topical PubMed search (B)		
**Decision Support Systems, Clinical [MeSH]** **Drug Therapy, Computer-Assisted [MeSH]** **Medical Order Entry Systems [MeSH]**		CPOE [Title/Abstract] Publication date from September 2009 to present		

**AND**				

**Alert optimization**		**Alert number**		**Alert response**
				
alert [Title/Abstract]		alert [Title/Abstract]		alert [Title/Abstract]
adopt/adopting/adoption		fatigue		compliant/compliance
specific/specificity		overload		override/overriding
prioritisation/prioritization		reduce/reducing/reduction		adherence/non-adherence
filter/filtering		over-alerting [Title/Abstract]		handling
improve/improving	**OR**		**OR**	user [Title/Abstract]
helpful				satisfaction
customize/customizing/customization				alert/user [Title/Abstract]
appropriate/inappropriate				acceptance
clinical/ly				response/responsiveness
significant/significance				human factors [Title/Abstract]
relevant/relevance				

MeSH terms are in bold.

• The specific search (A) used MeSH terms to search for CPOE-related articles.

• The topical search (B) on the general term "CPOE" discovered CPOE-related articles that have not been MeSH-indexed so far.

To reduce the number of hits, these two CPOE-related terms were combined with keywords regarding the alert optimization, alert number, and alert response (see Table 1).

Both researchers individually reviewed the abstracts of the found articles. Irrelevant papers were excluded if they did not contain any ideas that could be used for contextualized prioritization of CPOE alerts. All the relevant articles were included for full-text review and again read individually by both researchers. In order to extract possible context factors the researchers looked for correlations between the rates of overridden alerts and specific variables, for example, the number of years a doctor worked in the healthcare organization [19]. Furthermore, ideas for context factors were extracted if the authors of the papers suggested to tailor the alerts according to certain factors (for example, the complexity of the clinical case [20]) or aimed to increase the specificity and sensitivity of alerts by certain 'contextual' adaptations [12].

**Figure 3:**
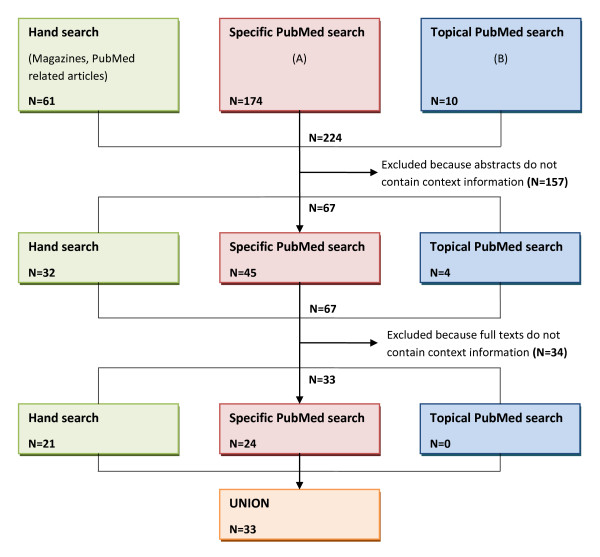
**Flowchart of literature search to elicit articles describing factors for the contextualization of CPOE alerts**. Due to overlaps between the three search strategies, the sums of papers in each line do not coincide with the number of papers provided after combining the results of the three searches.

A detailed flowchart of the performed literature search is shown in the following illustration (see Figure 3).

### Expert interviews

We performed qualitative interviews with leading international experts in the field of CPOE. We created a ranked list of experts on the basis of their number of publications as first named authors regarding CPOE-related papers (using the search terms "CPOE" in all fields and "medical order entry systems", "computer-assisted drug therapy", and "clinical decision support system" as major MeSH-headings within PubMed). First, we invited the authors with the most publications. We planned five interviews as a minimum and intended to conduct more interviews depending on the level of saturation, which means that data are collected until no new information can be retrieved [21]. In the end we conducted five interviews. Saturation was reached as the interviewed experts did not identify any new factors. When selecting the interview partners, we took care to ensure that none of them had any direct affiliation with the PSIP project.

The expert interviews were conducted using a semi-structured interview guideline. The interviewees were informed in advance by a description of our concept (cf. description of Figure 1) and the main interview question:

"Question: What could be the most important context factors that can be used for the prioritization of alerts?"

The telephone interviews were planned to take about 20 minutes. All the interviews were recorded and transcribed using summarized conversation protocols.

### Phase 2: Generation of context model

The extracted context factors from the literature search and the semi-structured interviews were generalized and hierarchically organized. To organize the factors we made use of an inductive category development according to Mayring [22]. Furthermore, for each context factor a generic definition and a specific example was generated. To collaboratively develop this context model we used an online brainstorming and mind map modeling tool.

### Phase 3: Validation of context model

The research team performed a first internal validation. Both researchers (MJ and DR) read all of the 33 full text articles that were found. Passages within those papers that indicated the contextualization of drug safety alerts were then independently classified using the final context model. The inter-researcher agreement for all text passages discovered by both researchers was determined by computing Cohen's Kappa coefficient. Disagreements in the classifications were put to discussion.

## Results

### The context model

The final context model is the result of the performed literature search and the five semi-structured expert interviews. In the interviews, five CPOE experts (having published between 3 and 13 CPOE-related articles on PubMed) agreed to participate (three from North America and two from Europe). They mentioned 12 possible context factors. In the literature search, 33 papers were identified that named 20 possible context factors, including the 12 factors named by the experts.

**Figure 4:**
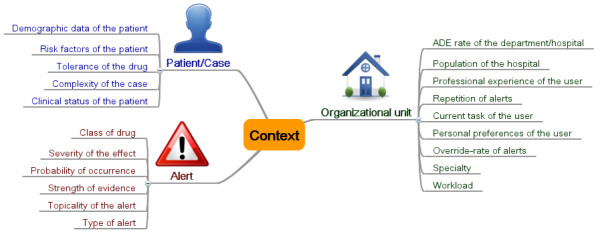
**Mind map of the final context model grouped in three categories**.

Table 2 lists all the identified context factors, together with an explanation. The found 20 context factors are grouped into three main categories (see also Figure 4).

**Table 2:** List of extracted context factors including definition and one example for each factor

	Definition	Example
	**ADE rate of the department/hospital** The total number of ADEs which occur in the department/hospital.	If a department/hospital has a low rate of a specific ADE, don't show the corresponding alerts.
	
	**Population of the hospital** The epidemiological characteristics of the patient population from the geographical catchment area of the hospital.	Show more alerts for increased risk of liver destruction when the prevalence for liver diseases is high in the area of the hospital.
	
	**Professional experience of the user** Years of working experience; degree and the position in the hierarchy.	A senior physician receives fewer alerts than a resident.
	
	**Repetition of alerts** The number of times a specific alert is presented to the user.	An alert is only shown to a doctor once a day or an alert is never shown twice for the same patient (e.g. after renewal of a prescription).
	
**Organizational unit**	**Current task of the user** The current step in the medication workflow (prescription, dispensation, administration).	Show all alerts during prescription, but only the most severe alerts during administration.
	
	**Personal preferences of the user** Individual customization of the alerts depending on the user's needs or preferences.	A doctor can turn off certain alerts if he or she doesn't want them.
	
	**Override-rate of alerts** The frequency a specific alert gets overridden by a specific user or department-/hospital-wide.	An alert won't be shown again to a doctor if he/she has already overridden it several times.
	
	**Specialty** The specialist field of the user or the special field of the department/hospital.	A psychiatrist receives different alerts than a surgeon.
	
	**Workload** The number of patients to care for, the staffing of the department, the duration of the shift or the time of the day.	Certain alerts that might be overlooked should be highlighted when the doctor is working for more than 8 h.

	**Demographic data of the patient** The sex, age and ethnicity of the patient.	Show certain alerts only for patients older than 60 years.
	
**Patient/Case**	**Risk factors of the patient** A certain genetic disposition, alcoholism, obesity or under-nutrition.	Show specific alerts only for alcoholics.
	
	**Tolerance of the drug** The case history of the patient shows that he/she tolerates a drug.	Don't show alerts for the possible side effects of aspirin, if the patient hasn't developed any of these in a previous case.
	
	**Complexity of the case** The number of clinical conditions and multi-morbidities of a patient or the number of applied drugs.	Certain alerts that might be overlooked should be highlighted in case that the patient has more than 5 chronic clinical conditions or if he takes more than 5 different drugs at the same time.
	
	**Clinical status of the patient** The type of disease, the severity or stadium of the disease or clinical parameters (e.g. lab values).	Show specific alerts only if the patient suffers from renal diseases or when a lab value reaches a critical threshold.

	**Class of drug** The group of the prescribed drug (e.g. narcotics, anticoagulants) relating to the possible damage it may cause.	Highlight specific alerts only for classes of drugs with a high ADE potential (e.g. corticosteroids).
	
**Alert**	**Severity of the effect** The seriousness of the potential effect.	Don't show alerts when the expected effect may cause no or only minor patient harm.
	
	**Probability of occurrence** The probability of occurrence of the expected ADE.	When prescribing an anticoagulant, show an alert only if the probability of internal bleeding is higher than 5%.
	
	**Strength of evidence** The strength of the scientific evidence of a certain effect.	Don't show alerts if only one non-randomized study reports this certain effect.
	
	**Topicality of the alert** How long a certain alert is in the system or the time since the last update of an alert.	Highlight alerts that are new in the system.
	
	**Type of alert** The type of drug interaction (e.g. drug-drug interaction, drug-allergy interaction) that may occur.	Don't show drug-allergy alerts if allergies are not sufficiently documented in the patient records of the hospital.

1. Characteristics of the organizational unit

Drug safety alerts can be prioritized based on the characteristics of the organizational unit. These context factors can affect three organization levels of health care: the entire hospital, a special hospital department, or an individual CPOE user. For example, certain types of alerts may be disabled for a specialized department, or a user with a lot of experience may want less intrusive alerts for a special group of drugs.

2. Characteristics of the patient/case

Information on the patient case can help to improve the tailoring of warnings. For example, recent lab values or known diagnoses may be taken into account when prioritizing alerts.

3. Characteristics of the alert

Warnings in CPOE systems can also be contextualized on the basis of alert characteristics. For example, alerts pointing to the possibility of less serious ADEs may be presented in a less intrusive way.

### Support for each context factor

From the 33 full text articles that were judged to be relevant in the literature search, the researchers identified an overall number of 133 text passages indicating the contextualization of drug safety alerts. Table 3 gives an overview of the number of text passages per context factors, with each passage either supporting or opposing a factor. Table 4 gives an overview of the 12 factors mentioned in the expert interviews.

**Table 3:** Literature support for each factor of the context model

	Context factor	Pros (+)	Cons (-)	N
	ADE rate of the department/hospital	[12,27]		2
	
	Population of the hospital	[15]		1
	
	Professional experience of the user	[12,15,20,23,24,27-31]	[12,19]	12
	
**Organizational unit**	Repetition of alerts	[11,12,14,20,27,29,31-35]		11
	
	Current task of the user	[11,14,19,33]		4
	
	Personal preferences of the user	[15,27,31,36,37]	[31,34]	7
	
	Override-rate of alerts	[11,15,31]		3
	
	Specialty	[11,12,15,23,27,28,38]	[35]	8
	
	Workload	[12,19,39]	[29]	4

	Demographic data of the patient	[12,19,20,27,29-31,37,38,40]	[29,35]	12
	
	Risk factors of the patient	[12,24]		2
	
**Patient/Case**	Tolerance of the drug	[12,15,20,31,35-37,40,41]		9
	
	Complexity of the case	[19,20,37,40]	[35]	5
	
	Clinical status of the patient	[12,19,27,29,33,35,37,38]		8

	Class of drug	[15,31,35,40,42]	[41]	6
	
**Alert**	Severity of the effect	[11,12,20,24,31,35,40,41,43-46]	[12,23,31,33,37,46]	18
	
	Probability of occurrence	[24,44]		2
	
	Strength of evidence	[11,20,24,27,33,40,47]		7
	
	Topicality of the alert	[28,41]		2
	
	Type of alert	[12,20,35,40,41,48-50]	[19,51]	10
	
			∑	**133**

"Pros" are references which positively discuss the given factor. "Cons" are references which negatively discuss the given factor. N gives the overall number of cases a factor has been discussed.

**Table 4:** Results of the quantitative content analysis of the semi-structured telephone interviews (N = 5 interviews)

Context factor	N
**Organizational unit**	

Personal preferences of the user	2

Specialty	2

Current task of the user	1

Override-rate of alerts	1

Repetition of alerts	1

**Patient/Case**	

Clinical status of the patient	4

Demographic data of the patient	4

Risk factors of the patient	1

Complexity of the case	1

Tolerance of the drug	1

**Alert**	

Severity of the effect	3

Strength of evidence	1

∑	**22**

N ... Number of interviewees who mentioned the context factor

### Internal model validation

Of those mutually found 133 text passages, 100 were individually found by both researchers (MJ and DR). For these 100 text passages, Cohen's Kappa was κ = 0.97 (for details see additional files).

## Discussion

### Answer to the study question

Based on a broad literature search and expert interviews, we identified 20 factors that could be used to contextualize drug safety alerts in CPOE systems. From the final 20 factors, 12 were mentioned during the expert interviews. Most factors that were named by more than one expert were also more intensively discussed in the literature (cf. Table 3 and Table 4). A possible explanation for this might be that there was an overlap between the authors of the papers found in the literature search and the CPOE experts invited for the telephone interviews (three out of five interviewees also appeared as authors of analysed papers).

### Strengths and weaknesses of the study

As strength, we combined interviews with a literature search during the development of the context model (cf. Figure 2). A literature search, which does not claim to be a systematic review, dominated the phase of factor extraction. We neither rated the type of article (RCT versus opinion paper) nor the section where the context information was gathered from (e.g. result vs. discussion). We cannot be sure that we identified all context factors ever discussed in the literature. However, as the interviewed experts did not identify other factors that we have found in the literature search, our list sufficiently reflects the state of scientific discussion at the moment.

The validation of the context model was conducted by the same two researchers who were involved in the model creation. This might explain the high Cohen's Kappa value of 0.97. Disagreements occurred in cases where the definitions of context factors only differ slightly. For instance, the definition of the factors 'repetition of alerts' (same alert shown frequently to the same user) and 'override-rate of alerts' (alerts overridden frequently by one user/all users from one department/all users from the hospital) only differ in the action the user takes when the alert is triggered (cf. Table 2). This could be seen in one non-agreement (see additional files).

We grouped the found 20 factors into three categories - naturally, other ways of groupings would be possible.

The final context model includes factors that differ in the level of abstraction. While the definitions of some context factors are very narrow (e.g. override-rate of alerts), others are more general (e.g. clinical status of the patient). This mostly reflects the different level of detail of the discussion in the literature, but also points to the need to limit the number of context factors by aggregating them.

No formal validation of completeness or external validation of the context model has been done yet.

### Results in relation to other studies

According to our knowledge, the developed context model is the first attempt to systematically reveal and structure 'context' that can be used for the prioritization of drug safety alerts. Some of the factors have been used within suggestions for alert improvement. For instance, van der Sijs et al. [23] (p.446) suggested that drug safety alerts might be improved if they were customized to professional experience, but also warned about turning off alerts for experts because of errors due to lack of attention, distraction, and forgetfulness. Sittig et al. [19], however, concluded in his study that there was no indication that users of different level of experience differed in their decisions to accept or ignore various CDS features. This example shows that tailoring alerts according to the professional experience is not consistently seen in the literature. A promising approach from our point of view is to tier alerts according to the level of severity which is, among others, also pursued by Paterno et al. [24]. She concluded that a tiered presentation of DDI alerts led to higher compliance rates of the physicians. A potential way to determine the level of severity of an alert is provided by the 'Medication Error Index' by the National Coordinating Council for Medication Error Reporting and Prevention, which provides a classification according to the severity of the outcome of a medication error [25]. Further studies are needed to better understand the feasibility of using each context factor for alert optimization, as well as the effect when using it.

### Meaning and generalizability of the study

The serious problem of alert overload causing alert fatigue and the ways to improve drug safety alerting in CPOE systems is a current issue [14].

Some of the presented context factors are already in use or may be considered in the development of future CPOE and CDS systems. For instance, within the PSIP project, the CDS system uses information on the diagnosis and lab values of a patient to trigger drug safety alerts.

However, for the implementation of several context factors into a real-world scenario, multiple preconditions have to be fulfilled in advance. For example, organizational (e.g. unique drug naming, international drug-interaction database, etc.), technical (e.g. data integration, standardization, etc.), and human (e.g. physicians' acceptance, etc.) aspects have to be taken into account.

The long-term experience with the Dutch drug database G-standard is now being used in the International Health Terminology Standards Development Organisation (IHTSDO) for the development of a good structure and unequivocal pharmaceutical information in SNOMED CT (Systematized Nomenclature of Medicine-Clinical Terms). In many countries, different commercial databases are used, and people are afraid of turning off alerts because of legal consequences. A shared international database could probably be used as a professional standard for those alerts that should be generated and those that can be turned off [23].

With respect to human factors, however, much is still unknown. It is unclear as to how the alerts of different severity and type (drug-interaction, duplicate order, or overdose) should be presented to the user (which colors, which form, which place on the screen, and whether visual or auditory signals should be used). The preferred order and length of the text components in the alert text is unknown, and which information should be available only in the background.

### Unanswered and new questions

The identified set of context factors for alert prioritization is proposed as a starting point for effective alerting in CPOE systems. At the moment, however, it is unclear which of the presented 20 context factors should be taken into account when triggering a drug safety alert. To address this unanswered question, a user survey among European physicians and a Delphi study among international CPOE experts has been conducted [26].

Another issue that should be dealt with in the future is the most effective combination of context factors. This could be subject of an international multi-disciplinary workshop of CPOE experts, users (physicians and pharmacists) as well as industry partners. The effectiveness should then be evaluated in subsequent experimental studies.

## Conclusion

To our knowledge, this is the first attempt to structure and validate possible context factors for alert optimization within CPOE systems. The ideas and concepts that have evolved throughout this work need further validation and clinical evaluation.

## Competing interests

The authors declare that they have no competing interests.

## Authors' contributions

DR and MJ carried out the literature search, performed the expert interviews, developed the context model, and accomplished the internal validation of the model. Furthermore, they drafted and revised the manuscript. WOH, WS and EA further refined the definitions and examples of the context factors. WOH and EA additionally participated in the development of the categories during the generation of the context model. WS and HS critically revised the manuscript and added an external point of view mainly in the discussion section. All authors read and approved the final manuscript.

## Pre-publication history

The pre-publication history for this paper can be accessed here:

http://www.biomedcentral.com/1472-6947/11/35/prepub

## Supplementary Material

Additional file 1 **List of hand-searched journals**. Click here for file

Additional file 2 **Cohen's Kappa for context model**. Inter-researcher agreement for 100 text passages indicating the contextualization of drug safety alerts that were individually found by the researchers. Click here for file
